# Mixed-species biofilms in food systems: from community assembly to ecology-informed management

**DOI:** 10.3389/fmicb.2026.1945500

**Published:** 2026-09-21

**Authors:** Francesca Coppola, Filomena Nazzaro, Florinda Fratianni, Flavio Polito, Jesus Fernando Ayala-Zavala, Vincenzo De Feo, Raffaele Coppola, Patrizio Tremonte

**Affiliations:** 1 Department of Agricultural Sciences, University of Naples “Federico II”, Portici, Italy; 2 Institute of Food Sciences, Italian National Research Council, Avellino, Italy; 3 Department of Pharmacy, University of Salerno, Fisciano, Italy; 4 Centro de Investigación en Alimentación y Desarrollo, A.C. (CIAD), Hermosillo, Mexico; 5 Department of Agricultural, Environmental and Food Sciences, University of Molise, Campobasso, Italy

**Keywords:** biofilm management, food safety, food-processing environments, microbial ecology, mixed-species biofilms

## Abstract

Biofilms in food-processing environments present multifaceted challenges for food safety, quality, and public health. These microbial communities, forming on a variety of surfaces within food-processing facilities, are responsible for persistent contamination, increased tolerance to cleaning and disinfection, and the transmission of spoilage organisms and pathogens. This review provides a comprehensive overview of current knowledge on the formation, ecological organization, persistence, and impact of mixed-species biofilms in food systems, emphasizing their central role in contamination control and food safety management. While recent advances in analytical technologies have improved our understanding of their complexity and resilience, significant knowledge gaps remain regarding their behavior under real-world industrial conditions. The review highlights the limitations of traditional eradication-based approaches and discusses innovative strategies for monitoring, controlling, and preventing biofilm-associated risks in modern food production systems.

## 1 Introduction

Microorganisms rarely exist as isolated populations in natural or engineered environments. Instead, they form structured communities in which ecological interactions shape survival, adaptation, and ecosystem functioning. Biofilms represent one of the predominant modes of microbial life and are widespread across natural, clinical, industrial, and food-associated environments. In food systems, microorganisms colonize both biotic and abiotic surfaces, becoming embedded within a self-produced extracellular matrix that promotes adhesion, spatial organization, nutrient retention, and protection against environmental stress. This lifestyle has profound implications for food safety, product quality, shelf life, and public health. Food-processing facilities provide numerous ecological niches that favor biofilm establishment, including food-contact surfaces, equipment junctions, conveyor belts, drains, gaskets, storage tanks, and utensils. Once established, biofilms are difficult to eradicate and may serve as persistent reservoirs of spoilage microorganisms and foodborne pathogens, thereby contributing to recurrent contamination in food-processing environments (Bridier et al., 2015; Flemming et al., 2016; Serrano et al., 2025). Biofilm-associated microorganisms generally display greater tolerance to environmental stresses, cleaning procedures, and disinfectants than their planktonic counterparts. Within biofilms, microbial cells undergo profound physiological and molecular adaptations, including altered metabolism, differential gene expression, phenotypic heterogeneity, and reduced susceptibility to antimicrobial treatments (Flemming et al., 2016). Consequently, biofilm-associated populations may survive sanitation procedures that effectively eliminate free-living cells, facilitating persistence, dispersal, and transmission throughout the food chain (Simões et al., 2010; Bridier et al., 2015). Beyond pathogen persistence, biofilms contribute to product spoilage, reduced shelf life, operational inefficiencies, and significant economic losses across multiple food sectors (Møretrø and Langsrud, 2017). Despite decades of research, much of our understanding of food-associated biofilms still derives from single-species models or simplified laboratory consortia. Although these systems have generated valuable mechanistic insights into adhesion, matrix production, maturation, and antimicrobial susceptibility, they only partially capture the taxonomic, spatial, functional, and environmental complexity of industrial food-processing environments, where multiple microbial populations coexist and interact under continuously changing conditions. Recent advances in high-resolution microscopy, omics technologies, imaging mass spectrometry, microfluidic platforms, and computational modeling now allow researchers to investigate community structure, spatial organization, functional heterogeneity, and temporal dynamics with unprecedented resolution, while also exposing the limited ecological realism of traditional reductionist models (Rodrigues et al., 2023; Coppola et al., 2025b).

For the purposes of this review, a mixed-species biofilm is defined as a surface-associated, spatially organized microbial community comprising two or more microbial species embedded, at least in part, within an extracellular matrix produced by members of the community. This operational definition distinguishes verified mixed-species biofilms from microbial assemblages whose spatial organization and biofilm-associated phenotype have not been directly demonstrated. Accordingly, facility microbiomes, surface-associated microbial communities, fermentation consortia, and microbial communities occurring within foods are not considered equivalent to mixed-species biofilms. Evidence from such systems is discussed only when it provides relevant ecological or mechanistic context for understanding community assembly, interactions, persistence, or functional outcomes in food-associated biofilms, and is identified as such where appropriate. These technological advances have supported a conceptual shift in the study of food-associated biofilms. Rather than being viewed as simple aggregates of individual microorganisms, biofilms are increasingly recognized as dynamic, spatially organized microbial communities whose properties emerge from complex ecological interactions. In food-processing environments, mixed-species biofilms have been documented across diverse processing settings and represent ecologically complex microbial communities. Their development, persistence, and responses to environmental disturbances are governed by cooperation, competition, interspecies communication, spatial organization, and environmental filtering, which collectively determine community assembly and ecosystem functioning (Røder et al., 2016; Flemming et al., 2016). Consequently, food safety-relevant traits – including pathogen persistence, spoilage potential, colonization resistance, sanitation tolerance, and recolonization dynamics – represent emergent community-level properties that cannot be reliably predicted from the behavior of individual species studied in isolation (Nadell et al., 2016; Møretrø and Langsrud, 2017). Throughout this review, however, persistence is interpreted according to the level of evidence available. At the community level, the term refers to the continued or recurrent presence of biofilm-associated populations or functions despite environmental disturbance or sanitation, whereas repeated detection of the same taxon over time is not considered evidence of persistence of the same strain. Strain-level persistence is considered established only when supported by longitudinal evidence based on sufficiently discriminatory typing or genomic approaches; otherwise, terms such as recurrent detection, repeated occurrence, or recolonization are preferred. Although not equivalent to surface-associated mixed-species biofilms, fermented-food microbial consortia provide experimentally tractable examples of relevant ecological principles, illustrating how microbial succession, metabolic complementarity, and interspecies interactions can shape community assembly and ecosystem functioning (Wolfe and Dutton, 2015). Viewing mixed-species biofilms through this ecological perspective may therefore complement pathogen-centered approaches by providing a community-level framework for understanding contamination dynamics and supporting food safety management (Burmølle et al., 2014; Flemming et al., 2016; EFSA Biohaz Panel, 2024).

Previous reviews have substantially advanced our understanding of mixed-species biofilms in food systems by addressing interspecies interactions, resistance to sanitation, analytical methodologies, and control strategies, while more recent contributions have emphasized their ecological organization or examined mixed biofilms within specific food sectors (Yuan et al., 2020; Sadiq et al., 2021; Kim et al., 2023; Ban-Cucerzan et al., 2025; Yuan et al., 2025). Building on these contributions, further integration of community assembly and ecological interactions with emergent functional outcomes, contamination and spoilage dynamics, environmental surveillance, risk assessment, and ecology-informed management may provide a more unified perspective on mixed-species biofilms in food systems. The present review seeks to complement previous syntheses by organizing available evidence along this ecological continuum, from environmental filtering and community assembly to predictive and context-specific management strategies. Table 1 compares the scope, evidence base, limitations, and distinctive contribution of the present review with selected previous syntheses. Against this background, we examine mixed-species biofilms in food systems through an ecological lens, integrating community assembly and interaction dynamics with practical aspects of contamination control and food safety management. We discuss the mechanisms governing community assembly, ecological interactions, and biofilm persistence, together with the technological advances that have transformed our understanding of these complex microbial communities. We then examine how these ecological processes influence contamination dynamics, spoilage, and pathogen persistence across food environments before reviewing current and emerging strategies for biofilm monitoring, prevention, and control. Finally, we highlight future research priorities for translating ecological knowledge into measurable, validated, and context-specific approaches to biofilm monitoring, risk assessment, and management.

**TABLE 1:** Comparison of the scope, evidence base, scope boundaries, and distinctive contribution of selected previous reviews and the present review on mixed-species biofilms in food systems.

Review	Main scope	Evidence base / systems considered	Scope boundaries / limitations	Distinctive contribution
Yuan et al. (2020)	Mixed-species biofilms in the food industry, with emphasis on microbial interactions, resistance, detection, and control.	Experimental evidence on mixed-species biofilms relevant to food safety and quality.	Broad coverage of mixed-biofilm biology and control; community assembly and its translation into ecology-informed management were outside its principal focus.	Integrated overview of social interactions in mixed-species biofilms and their implications for resistance, detection, and control.
Sadiq et al. (2021)	Ecological organization and behavior of multispecies biofilms in the food industry.	Studies addressing multispecies biofilms and ecological interactions in food-related settings.	Primarily focused on understanding multispecies-biofilm ecology rather than extending the ecological framework to surveillance, risk assessment, and management.	Explicitly positioned multispecies food biofilms within an ecological perspective and highlighted the importance of community interactions.
Kim et al. (2023)	Methodological approaches for investigating mechanisms in multispecies biofilms involving foodborne pathogens.	Experimental multispecies models addressing spatial distribution, microbial interactions, EPS production, and quorum sensing.	Deliberately methodological and mechanistic in scope, rather than designed as a food-system-level synthesis.	Detailed framework of experimental approaches for elucidating mechanisms underlying multispecies-biofilm behavior.
Ban-Cucerzan et al. (2025)	Biofilm formation, food safety implications, detection, control, and economic impact in food-processing environments.	Broad literature on food-industry biofilms, including biological mechanisms, detection, intervention, and economic aspects.	Broad biofilm-centered perspective encompassing multiple dimensions; community assembly and ecological processes are not used as the main organizing continuum.	Comprehensive synthesis connecting biofilm biology with detection, control, regulatory considerations, and economic implications.
Yuan et al. (2025)	Characteristics, microbial interactions, and control of mixed biofilms in dairy processing.	Evidence specifically related to mixed biofilms and microbial communities in dairy-processing systems.	Intentionally sector-specific, providing detailed coverage of dairy environments.	In-depth synthesis of mixed-biofilm composition, social interactions, properties, and control within dairy processing.
Present review	Mixed-species biofilms in food systems viewed across the continuum from community assembly to ecology-informed management.	Evidence from verified surface-associated mixed-species biofilms, complemented, where explicitly identified, by relevant ecological evidence from experimental consortia, facility communities, and food-associated microbiota.	Narrative rather than systematic synthesis; available evidence is heterogeneous across food sectors, experimental models, and industrial settings.	Complements previous syntheses by integrating environmental filtering and community assembly with ecological interactions, emergent functional outcomes, contamination and spoilage dynamics, surveillance, risk assessment, and predictive, ecology-informed management within a unified conceptual framework.

This article was conceived as a narrative review aimed at critically integrating current knowledge on mixed-species biofilms in food-related environments rather than providing a systematic or quantitative synthesis of the literature. Relevant studies were identified through searches of major scientific databases, including PubMed, Scopus, and Web of Science, using combinations of terms related to “mixed-species biofilm”, “multispecies biofilm”, “food processing”, “food industry”, “food safety”, “microbial interactions”, “biofilm persistence”, and “biofilm control”. Particular attention was given to recent peer-reviewed studies addressing mixed-species biofilms under food-relevant conditions, while complementary evidence from other microbial community systems was considered when relevant to the ecological or mechanistic aspects discussed. Earlier seminal studies were retained when necessary to provide mechanistic or conceptual context. Throughout the review, evidence is interpreted according to its experimental and translational context, distinguishing naturally occurring from experimentally constructed communities and laboratory-scale models from pilot-scale and industrial observations. Evidence derived from non-food biofilm systems or theoretical models is used only when it provides relevant mechanistic or conceptual support and is identified accordingly. Because the literature search was narrative rather than systematic, the evidence discussed herein should not be interpreted as providing quantitative estimates of prevalence or as an exhaustive representation of all published studies. Figure 1 summarizes this conceptual framework, illustrating how environmental filtering and community assembly shape ecological interactions, emergent community properties, and management strategies throughout the review.

**FIGURE 1:**
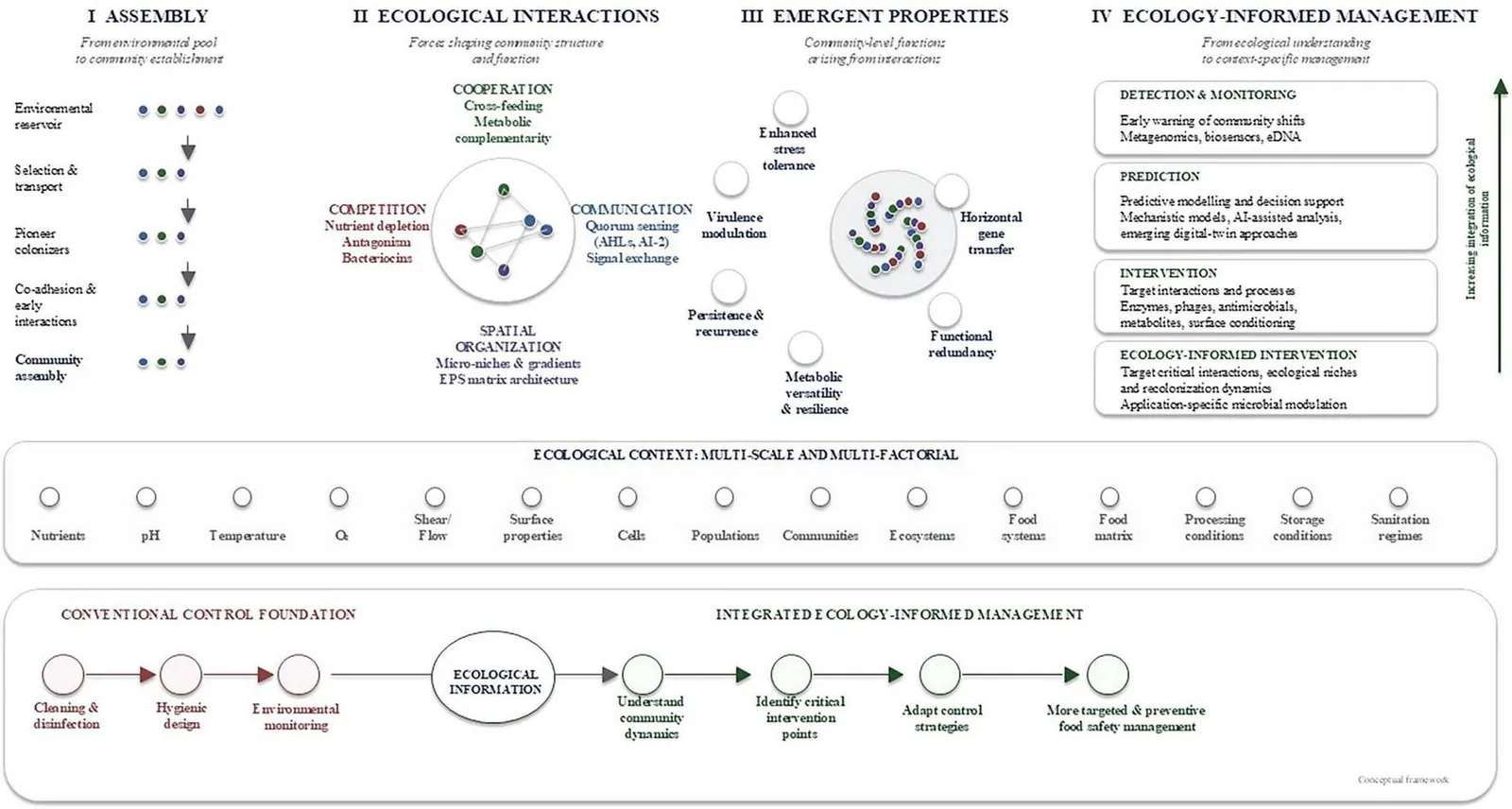
Ecological framework of mixed-species biofilms in food systems: from community assembly to ecology-informed management.

## 2 Formation and occurrence of mixed-species biofilms in food systems

Mixed-species biofilms arise through dynamic community assembly processes rather than from random microbial accumulation. Their composition and organization are shaped by microbial traits, environmental conditions, dispersal, colonization history, and the physicochemical properties of available surfaces. Biofilm development involves initial colonization, recruitment, community succession, structural reorganization, and dispersal, with trajectories that may vary according to environmental conditions and disturbance. Understanding these assembly processes is essential for identifying the mechanisms underlying biofilm persistence and recurrent contamination in food-processing environments.

### 2.1 Occurrence and prevalence in food environments

Mixed-species biofilms have been documented across different stages and sectors of the food-production chain and represent an important form of microbial organization in food-processing environments (Yuan et al., 2025). Naturally occurring communities may exhibit considerable taxonomic and functional diversity, with their composition influenced by food residues, environmental conditions, sanitation practices, dispersal events, and interspecies interactions (Røder et al., 2016; Kim et al., 2023). Food-processing facilities provide conditions conducive to biofilm formation, including intermittent nutrient availability, persistent moisture, temperature fluctuations, and protected niches that may evade routine cleaning. Consequently, biofilms can develop on both food-contact and non-food-contact surfaces, including stainless-steel equipment, conveyor belts, storage tanks, seals, plastic components, and floor drains, where they may persist despite regular sanitation (Møretrø and Langsrud, 2017).

Biofilm establishment is also influenced by surface characteristics and by food-derived conditioning films that modify microbial attachment (Whitehead and Verran, 2015). The composition and occurrence of mixed-species biofilms vary among food sectors and processing environments. Meat-processing environments frequently harbor spoilage microorganisms, environmental bacteria, and foodborne pathogens, whereas fresh-produce systems contain diverse plant-associated microbiota that may influence pathogen persistence and product deterioration (Wagner et al., 2020; Nikolaev et al., 2022; Calhoun et al., 2025; Brandl, 2006; Olaimat and Holley, 2012; Sheng and Zhu, 2021; Arthur and Gil, 2025; Zaman et al., 2025). Dairy environments may include starter and non-starter lactic acid bacteria, spoilage microorganisms, and potential pathogens, while seafood-processing environments commonly harbor psychrotrophic microorganisms associated with spoilage and contamination (Bintsis and Kyritsi, 2025). Fermented foods and beverages likewise support complex microbial consortia that, although not equivalent to surface-associated mixed-species biofilms, provide relevant examples of microbial interactions associated with desirable fermentation or spoilage. Table 2 summarizes representative taxa, colonization niches, and ecological processes across major food-production sectors.

**TABLE 2:** Ecological characteristics of mixed-species biofilms across major food-production sectors.

Food-production sector	Representative microbial groups, colonization niches, and ecological features	Representative references
Meat and meat products	Representative microbial groups: *Pseudomonas* spp., *Brochothrix thermosphacta*, lactic acid bacteria (LAB), Enterobacteriaceae, *Listeria monocytogenes*, and other resident environmental bacteria. Representative niches: conveyor belts, slicers, knives, deboning tables, equipment junctions, drains, and chilling rooms. Representative ecological features: pioneer colonization, ecological succession, nutrient competition, matrix-mediated protection, metabolic interactions, and context-dependent associations between resident microbiota, spoilage organisms, and foodborne pathogens.	Tremonte et al., 2017; Wagner et al., 2020; Nikolaev et al., 2022; Calhoun et al., 2025
Dairy	Representative microbial groups: *Pseudomonas* spp., *Microbacterium* spp., *Stenotrophomonas* spp., LAB, yeasts, staphylococci, and *Listeria monocytogenes*. Representative niches: milking systems, pipelines, pasteurizers, cheese vats, storage tanks, drains, and milk-contact surfaces. Representative ecological features: co-adhesion, matrix production and sharing, metabolic complementarity, ecological succession, and coexistence of technological, spoilage-associated, and potentially pathogenic populations.	Fox et al., 2017; Bintsis and Papademas, 2024; Diaz et al., 2025
Fresh produce	Representative microbial groups: Enterobacteriaceae, *Pseudomonas* spp., LAB, plant-associated microorganisms, phytopathogens, *Salmonella enterica*, pathogenic *Escherichia coli*, and *Listeria monocytogenes*. Representative niches: leaf and fruit surfaces, washing systems, flumes, cutting equipment, irrigation lines, and water-recirculation units. Representative ecological features: environmental filtering, plant–microbe interactions, ecological succession, competitive exclusion, interspecies signaling, and context-dependent pathogen persistence.	Brandl, 2006; Sheng and Zhu, 2021; Arthur and Gil, 2025
Seafood	Representative microbial groups: *Pseudomonas* spp., *Shewanella* spp., *Photobacterium phosphoreum*, LAB, and, in some processing environments, *Listeria monocytogenes*. Representative niches: ice machines, filleting equipment, cold rooms, storage facilities, drains, and food-contact surfaces exposed to chilled seawater or processing residues. Representative ecological features: low-temperature selection, psychrotrophic adaptation, metabolic interactions, oxygen-dependent niche differentiation, ecological succession, and stress tolerance.	Gram and Dalgaard, 2002; Parlapani et al., 2014; Parra-Flores et al., 2022; Wu et al., 2026
Beverages and fermented foods	Representative microbial groups: LAB, acetic acid bacteria, yeasts, molds, and environmental bacteria. Representative niches: fermentation vessels, maturation surfaces, bottling lines, filling nozzles, pipelines, and storage tanks. Representative ecological features: ecological succession, metabolic complementarity, interspecies signaling, competition, and coexistence of desirable technological microbiota and spoilage-associated populations.	Srey et al., 2013; Iarusso et al., 2025

### 2.2 Mechanisms governing initial attachment and co-adhesion

Mixed-species biofilm formation begins with the attachment of pioneer microorganisms to biotic or abiotic surfaces. In food-processing environments, successful colonization depends on microbial traits, surface properties, transport dynamics, and local environmental conditions. Initial attachment is generally reversible and governed by physicochemical interactions, while food-derived conditioning films composed of proteins, lipids, polysaccharides, and minerals can modify surface properties and favor microbial colonization (Bos et al., 1999; Whitehead and Verran, 2015; Giaouris et al., 2014). Microbial appendages, including flagella, pili, fimbriae, curli fibers, and adhesins, subsequently promote stable adhesion, while extracellular polymeric substance (EPS) production contributes to cell association, nutrient retention, and protection against environmental stresses and sanitation procedures (Flemming et al., 2016).

Co-aggregation and co-adhesion further contribute to multispecies community assembly. Co-aggregation occurs between planktonic microorganisms, whereas co-adhesion involves the attachment of suspended cells to microorganisms already associated with a surface. These processes facilitate species recruitment and community organization, with some microorganisms acting as bridging organisms between otherwise weakly associated populations (Rickard et al., 2003). Pioneer colonizers may therefore influence subsequent community development through priority effects and niche modification. In food-processing environments, *Pseudomonas* spp. frequently act as early colonizers and may facilitate the establishment of spoilage microorganisms and foodborne pathogens, whereas lactic acid bacteria can influence community succession by occupying attachment sites and co-aggregating with other microorganisms (Wagner et al., 2020; Calhoun et al., 2025). Thus, initial attachment and early microbial interactions can strongly influence subsequent community composition and biofilm development.

### 2.3 Determinants of community assembly and biofilm development

Following initial colonization, mixed-species biofilm development is shaped by environmental filtering, microbial interactions, succession, dispersal, priority effects, stochastic processes, and recurrent disturbance. Consequently, communities exposed to similar processing conditions may follow different trajectories depending on colonization history and local environmental conditions.

In food-processing environments, temperature, pH, water activity, nutrient and oxygen availability, hydrodynamics, surface characteristics, and food residues act as selective pressures on community composition. Recurrent disturbances, particularly cleaning and disinfection, may further remove susceptible populations while favoring stress-tolerant microorganisms and subsequent recolonization, potentially contributing to the establishment of persistent biofilms (Møretrø and Langsrud, 2017; Fagerlund et al., 2017).

Community assembly reflects both deterministic processes, such as environmental selection and niche differentiation, and stochastic processes including dispersal, ecological drift, and historical contingency (Zhou and Ning, 2017). Pioneer populations can further modify local conditions through nutrient consumption, metabolite production, oxygen depletion, matrix formation, and surface conditioning, thereby influencing subsequent colonization. Repeated cycles of colonization, growth, detachment, and recolonization may facilitate dissemination to new surfaces and contribute to recurrent contamination within processing facilities (Flemming et al., 2016; Carpentier and Cerf, 2011). Together, these processes influence whether mixed-species communities remain transient or develop more stable and persistent structures (Luo et al., 2022), thereby providing the ecological context for the microbial interactions discussed in Section “3 Ecological interactions in mixed-species biofilms”.

## 3 Ecological interactions in mixed-species biofilms

Mixed-species biofilms function as dynamic microbial ecosystems whose behavior emerges from interactions among constituent microorganisms and their environment (Tepson and Agyirifo, 2026). As illustrated in Figure 2, cooperation, competition, communication, and spatial organization interact to shape community structure and function, potentially influencing physiological heterogeneity, metabolic complementarity, colonization resistance, and persistence. The outcomes of these interactions are strongly dependent on community composition and environmental conditions, making their evaluation particularly relevant for understanding the behavior of mixed-species biofilms in food-processing environments.

**FIGURE 2:**
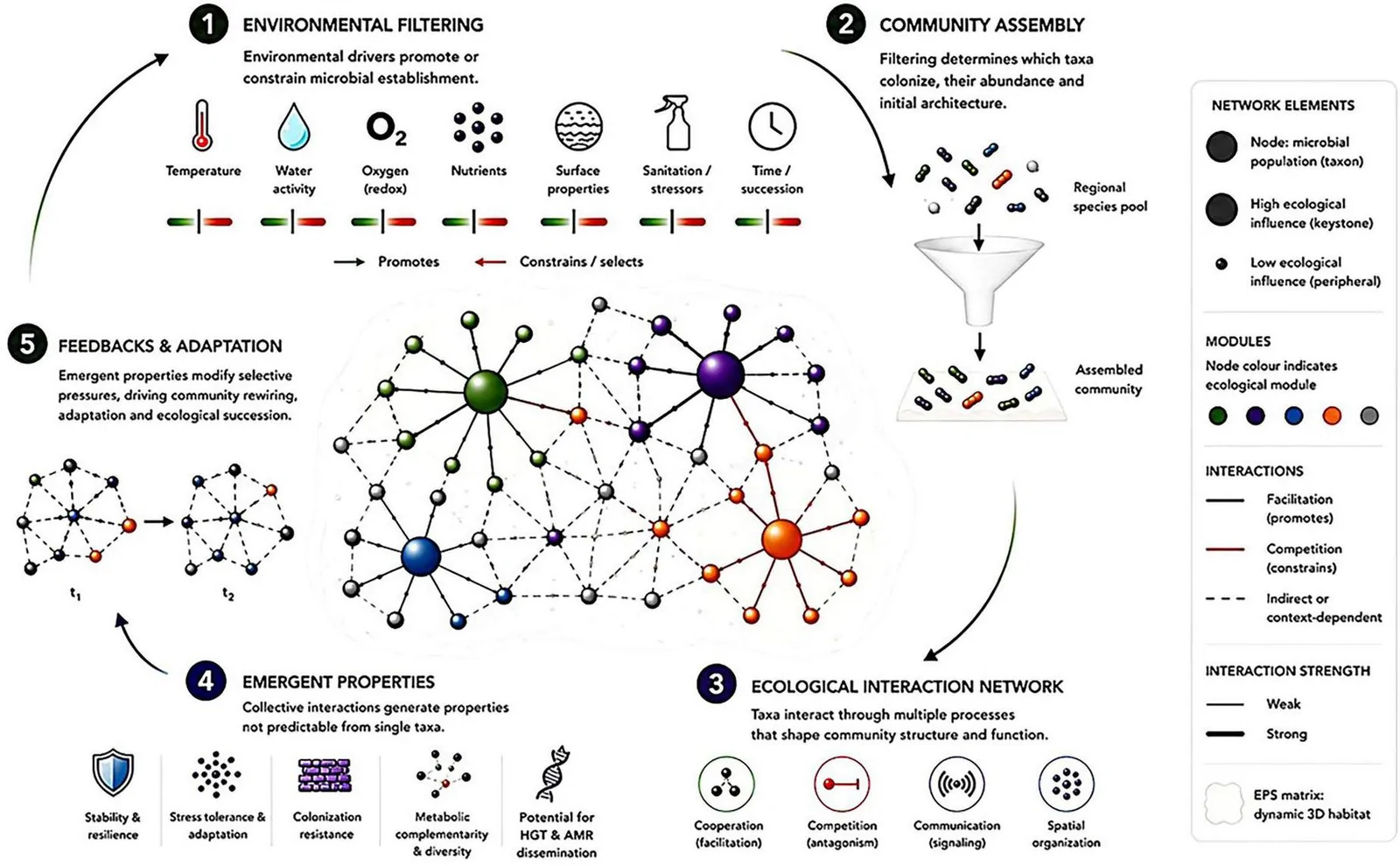
Ecological framework governing the assembly, organization, and persistence of mixed-species biofilms.

### 3.1 Cooperative interactions and synergistic relationships

Cooperative and facilitative interactions can influence the establishment and persistence of mixed-species biofilms (Yao et al., 2022). In food-processing environments, fluctuating nutrient availability, sanitation, temperature variation, osmotic stress, and desiccation may favor interactions that improve resource acquisition, matrix development, stress tolerance, or niche exploitation. However, these effects are strongly context dependent and frequently coexist with competition and exploitation.

Co-adhesion and co-aggregation represent early mechanisms of community assembly, whereby pioneer colonizers can provide attachment sites for secondary populations through surface-associated structures and extracellular polymers (Rickard et al., 2003). In food-processing environments, early colonizers may therefore facilitate the incorporation of spoilage microorganisms or foodborne pathogens, although these effects depend on species composition, colonization history, and environmental conditions (Liu et al., 2016; Wagner et al., 2020; Calhoun et al., 2025).

Metabolic cross-feeding can facilitate coexistence when metabolites released by one population support neighboring microorganisms, particularly where close spatial organization favors metabolite exchange. Such interdependence may, however, become disadvantageous when key populations are lost or environmental conditions change. Similarly, siderophores may act as shared resources or promote competitive sequestration depending on receptor compatibility and community composition. Although experimental and computational studies support important roles for metabolite exchange in microbial communities, direct evidence from food-associated mixed-species biofilms remains comparatively limited (Valiei et al., 2024).

Functional complementarity may broaden community capabilities through contributions to nutrient transformation, matrix production, detoxification, or stress protection, while functional redundancy may increase robustness when populations perform overlapping functions. The EPS matrix can likewise act as a partially shared structural and protective resource, enhancing attachment, hydration, nutrient retention, or protection of neighboring microorganisms. However, these benefits depend on community composition and spatial organization and may also create opportunities for exploitation (Flemming et al., 2016; Nadell et al., 2016; Flaig et al., 2026).

Evidence from food-associated systems illustrates the context dependence of these interactions. In dairy environments, resident microbiota and lactic acid bacteria can influence pathogen colonization through matrix production, metabolic interactions, antimicrobial activity, and competition for ecological niches (Gómez et al., 2016). In meat-processing systems, *Pseudomonas* spp. and other spoilage-associated microorganisms may either facilitate or restrict foodborne pathogens depending on community composition (Wagner et al., 2020; Calhoun et al., 2025). Defined multispecies experiments have likewise shown that particular strain combinations can generate greater biofilm biomass than corresponding single-species cultures, although these responses are strain-specific (Sadiq et al., 2023a). Thus, cooperation can contribute to community stability and persistence, but increased resilience should not be regarded as an intrinsic property of multispecies growth.

### 3.2 Competitive interactions and antagonism

Competitive interactions are important determinants of community assembly and stability. Microorganisms compete for nutrients, attachment sites, electron acceptors, micronutrients, and ecological niches, with outcomes depending on community composition and environmental conditions. Competition may occur through differential resource acquisition or through direct interference with neighboring populations.

During biofilm formation, differences in resource availability can alter microbial dispersal, relative abundance, and community assembly, thereby influencing subsequent community structure (Jeong and Kim, 2025). Competition for iron is one example, as siderophore production under iron limitation can either facilitate iron sharing or restrict access by competing populations depending on receptor compatibility (Butaitë et al., 2017).

Interference competition includes the production of organic acids, bacteriocins, biosurfactants, and other inhibitory metabolites. Such mechanisms are particularly relevant to lactic acid bacteria, which can suppress pathogens and spoilage microorganisms in food-associated systems (Silva et al., 2018). However, antimicrobial effects observed in planktonic cultures cannot be assumed to occur to the same extent within structured multispecies biofilms, where matrix organization, diffusion, spatial segregation, and physiological heterogeneity may alter exposure and susceptibility.

Direct contact-dependent mechanisms, including Type VI secretion systems (T6SS), contact-dependent inhibition (CDI), and polymorphic toxins, may also contribute to antagonism; however, their ecological importance in food-processing biofilms remains poorly resolved, and current evidence derives largely from non-food systems.

Competition may also involve interference with microbial signaling through quorum-quenching mechanisms, although their effects depend strongly on the signaling systems and microorganisms involved (Grandclément et al., 2015; Wang et al., 2023; Ding et al., 2026).

Competitive interactions can also contribute to colonization resistance, as established communities may restrict invasion by occupying attachment sites, depleting nutrients, producing inhibitory metabolites, or modifying local physicochemical conditions. Such resistance remains dynamic, however, because environmental disturbance can create opportunities for better-adapted populations to invade or replace resident microorganisms.

Food-associated evidence illustrates both the relevance and context dependence of these processes. In dairy systems, lactic acid bacteria can inhibit pathogens and spoilage microorganisms through nutrient competition, acidification, and bacteriocin production (Gómez et al., 2016; Silva et al., 2018). In meat-processing environments, resident and spoilage-associated microbiota may influence pathogen establishment through competition for resources and attachment sites (Wagner et al., 2020; Ban-Cucerzan et al., 2025). Observations from ready-to-eat facilities similarly indicate associations between resident community composition and pathogen establishment or recurrent detection, although community-level observational data generally cannot identify the specific competitive mechanisms involved (Diaz et al., 2025).

### 3.3 Communication networks and interspecies signaling

Microbial communication can influence attachment, matrix production, resource utilization, stress responses, maturation, and dispersal within biofilms (Xu et al., 2026). Quorum sensing (QS), the best-characterized signaling mechanism in bacterial biofilms, regulates multiple biofilm-associated traits through the production and perception of extracellular autoinducers (Das et al., 2026; Ding et al., 2026). QS systems differ among microorganisms, with many Gram-negative bacteria using N-acyl homoserine lactones (AHLs) and Gram-positive bacteria commonly employing autoinducing peptides (AIPs). In food-associated microorganisms, including *Pseudomonas*, *Aeromonas*, *Serratia*, *Listeria*, *Enterococcus*, and some lactic acid bacteria, QS has been associated with biofilm development, spoilage activity, virulence, and environmental adaptation (Bai and Rai, 2011; Skandamis and Nychas, 2012; Mukherjee and Bassler, 2019).

Autoinducer-2 (AI-2), produced through the LuxS-dependent pathway by numerous Gram-positive and Gram-negative bacteria, has been widely investigated as a potential mediator of interspecies signaling. Importantly, AI-2 production alone does not demonstrate communication; evidence of signal perception and a measurable response in neighboring populations is required. Experimental studies have associated AI-2 with biofilm development, matrix production, and community organization, although its effects remain strongly species and context dependent (Pereira et al., 2013; Jiang et al., 2022).

Communication may also involve metabolite-mediated signaling and outer membrane vesicles (OMVs), which can contribute to the exchange of signaling molecules and other bioactive compounds within structured microbial communities (Toyofuku et al., 2019).

In food-associated systems, signaling may influence interactions among starter cultures, resident microbiota, spoilage microorganisms, and pathogens, with potential consequences for biofilm development, spoilage-associated metabolism, stress responses, and virulence (Bai and Rai, 2011; Skandamis and Nychas, 2012; Sadiq et al., 2023b). However, much of the mechanistic evidence for interspecies signaling derives from defined experimental systems, and its contribution to naturally occurring industrial mixed-species biofilms remains less well established. This knowledge has nevertheless stimulated interest in quorum quenching as a complementary control strategy, as discussed in Section “5 Control and management of mixed-species biofilms” (Grandclément et al., 2015; Miguel et al., 2026).

### 3.4 Spatial organization and emergent properties of multispecies biofilm communities

The three-dimensional architecture of mixed-species biofilms both shapes and is shaped by microbial interactions. Spatial variation in cellular composition, matrix organization, nutrient and oxygen availability, and local physicochemical conditions influences microbial proximity, metabolite exchange, signaling, and exposure to environmental stress. Spatial segregation may reduce competition and promote niche differentiation, whereas proximity can facilitate cross-feeding, signaling, and matrix sharing but may also intensify competition or antagonism. Thus, the functional consequences of spatial organization depend on community composition and environmental conditions (Liu et al., 2019; Dal Co et al., 2023; Bäcker et al., 2026).

Biofilm architecture also influences responses to sanitation. Dense or chemically reactive matrices can retard, bind, or inactivate disinfectants, while physicochemical gradients and physiological heterogeneity generate populations with different susceptibilities. Survival following sanitation may therefore reflect interacting mechanisms – including matrix properties, transport limitation, physiological state, and post-treatment recovery – rather than impaired disinfectant penetration alone (Bridier et al., 2011; Flemming et al., 2016).

Spatial organization may contribute to resilience through protected microniches, metabolic complementarity, and physiological heterogeneity, although increased tolerance is not universal and antagonistic interactions may instead increase susceptibility (Dal Co et al., 2023; Bäcker et al., 2026). Resilience should therefore be demonstrated experimentally rather than inferred from greater taxonomic complexity alone.

Repeated disturbances may induce persistent changes in community composition and interaction patterns, sometimes conceptualized as ecological memory. However, evidence for this phenomenon in food-processing biofilms remains limited, and it should be distinguished from selection of stress-tolerant populations or cellular stress memory and regarded as a testable hypothesis rather than an established property of industrial multispecies biofilms.

Industrial studies have documented recurrent or long-term detection of multispecies communities in dairy, meat, seafood, and ready-to-eat facilities, but community detection does not necessarily demonstrate a biofilm-associated phenotype or resolve three-dimensional organization. For example, Diaz et al. (2025) characterized microbial community dynamics in a ready-to-eat facility with long-term L. monocytogenes colonization without directly resolving community spatial architecture. Species-resolved imaging combined with longitudinal genomic, transcriptomic, and environmental analyses will therefore be important for determining whether and how biofilm architecture contributes to persistence and sanitation failure under industrial conditions.

Overall, evidence ranges from mechanistic studies using defined laboratory consortia to reconstructed food-associated communities and direct industrial observations. These approaches provide complementary evidence, with controlled systems offering greater mechanistic resolution and industrial studies greater ecological realism but more limited causal inference. Table 3 summarizes the principal ecological mechanisms discussed in this section, while Table 4 compares representative primary studies across different levels of experimental and translational evidence.

**TABLE 3:** Ecological processes governing mixed-species biofilms and their implications for food systems.

Ecological process	Main mechanisms	Ecological significance	Implications for food systems
Cooperation and facilitation	Co-adhesion, metabolic cross-feeding, extracellular matrix production and sharing, functional complementarity	Can enhance partner recruitment, resource-use efficiency, stress protection and community integration; may also generate metabolic interdependence	Increased biofilm persistence, enhanced spoilage potential, reduced sanitation efficacy and, in some communities, facilitation of pathogen establishment
Competition and antagonism	Competition for nutrients and attachment sites, siderophore-mediated iron acquisition, bacteriocins, organic acids, contact-dependent inhibition (CDI), Type VI secretion system (T6SS), interference competition	Regulates species composition, niche occupation, ecological succession and colonization resistance	Pathogen exclusion or persistence depending on community composition; selection of resident microbiota; altered contamination dynamics
Communication and signalling	Quorum sensing (AHLs, AIPs and AI-2), metabolite-mediated signalling, signal interception, quorum quenching, outer membrane vesicles (OMVs)	Coordinates collective behaviours, biofilm maturation, stress responses and community adaptation	Modulation of spoilage activity, virulence, persistence and susceptibility to communication-targeted control strategies

EPS, extracellular polymeric substances; AHLs, N-acyl homoserine lactones; AIPs, autoinducing peptides; AI-2, autoinducer-2; eDNA, extracellular DNA; OMVs, outer membrane vesicles; T6SS, Type VI secretion system; HGT, horizontal gene transfer. Representative references: Flemming et al. (2016); Nadell et al. (2016); Røder et al. (2016); Mukherjee and Bassler (2019); Sadiq et al. (2023a) 2023b; Butaitë et al. (2017); Valiei et al. (2024); Jeong and Kim (2025); Sadiq et al. (2026); Bäcker et al. (2026).

**TABLE 4:** Representative evidence supporting current knowledge of mixed-species biofilms in food systems across experimental and industrial scales.

Study	Food system / biofilm model	Evidence type & scale	Experimental design / intervention	Main findings & translational considerations
Wagner et al., 2020	Meat-processing environment; food-contact and non-food-contact surfaces; naturally occurring multispecies biofilms containing spoilage-associated bacteria.	Natural community; industrial *in situ* evidence.	Factory surfaces were sampled and biofilm hotspots characterized using microbial detection together with matrix-associated biofilm evidence; no experimental intervention.	Verified multispecies biofilms were identified directly in the processing environment, supporting high industrial relevance. Observational design is strong for occurrence and localization but does not establish causal interspecies interactions.
Nikolaev et al., 2022	Meat-processing plant; biofilms collected from industrial surfaces and characterized for microbial composition and structure.	Natural community; industrial observational evidence.	Environmental biofilm samples were examined using culture-independent/community profiling and microscopy-based structural characterization; no intervention comparator.	Demonstrates taxonomically complex biofilm communities under real processing conditions. Industrial sampling increases translational relevance, whereas observational data provide limited mechanistic resolution.
Diaz et al., 2025	Ready-to-eat food-production facility with long-term Listeria monocytogenes colonization; environmental surface microbiota.	Natural surface community; industrial longitudinal/observational evidence.	Environmental samples from different factory areas were profiled over time to examine microbial composition, dynamics, and coexistence with Listeria spp.; no intervention.	Shows stable, area-specific facility microbiota and recurrent Listeria detection. Highly relevant industrially, but community profiling does not by itself demonstrate spatially organized mixed-species biofilm architecture or strain-level persistence.
Fagerlund et al., 2017	Meat-processing conveyor-belt model; *L. monocytogenes* with multigenus background microbiota isolated from meat-processing surfaces.	Constructed multigenus community from industrial isolates; food-relevant laboratory model.	Biofilms on conveyor-belt coupons at 12 °C were exposed to repeated cleaning/disinfection cycles using chlorinated alkaline cleaning followed by QAC- or PAA-based disinfectants; mono-/multigenus conditions provided comparators.	Reproduced survival and community shifts under industry-relevant cleaning conditions and showed spatial intermixing of L. monocytogenes with background flora. Strong bridge between field isolates and controlled experimentation, but still a reconstructed laboratory community.
Sadiq et al., 2023a	Dairy, meat, and egg-processing isolates; four-species biofilm combinations, including validation on stainless steel.	Constructed communities from industrial food-contact isolates; laboratory scale.	Ninety-two strains were screened and 455 four-species combinations compared with corresponding monocultures; biofilm biomass and selected community structure were evaluated.	Identified strain-specific synergy and candidate keystone taxa, particularly among dairy isolates. Broad combinatorial testing supports interaction hypotheses, but simplified four-species communities cannot reproduce full industrial diversity.
Sadiq et al., 2023b	Dairy pasteurizer isolates: *Stenotrophomonas rhizophila*, *Bacillus licheniformis*, *Microbacterium lacticum*, and *Calidifontibacter indicus*; stainless-steel biofilm model.	Constructed four-species community from industrial isolates; mechanistic laboratory scale.	Pairwise, three-species, and four-species combinations were compared in BHI and, for selected analyses, skim milk; biomass, viable-cell dynamics, SEM, and cell-free-supernatant effects were assessed.	Resolved commensalism, exploitation, amensalism, higher-order interactions, and a keystone role for M. lacticum. Mechanistically informative but based on a deliberately simplified community under controlled conditions.
Liu et al., 2016	Fresh-produce-processing relevance; *Ralstonia insidiosa* FC1138 from a processing plant combined separately with *L. monocytogenes*, *Salmonella enterica*, and enterohemorrhagic *Escherichia coli*.	Constructed dual-species communities using an industrial environmental isolate plus pathogen strains; laboratory scale.	Dual-species biofilms were compared with monocultures; biomass/pathogen incorporation, cell-cell contact, surface priming, and metabolite-related mechanisms were examined under controlled culture conditions.	Supports a bridging role for *R. insidiosa* in pathogen incorporation into biofilms. Food-industry origin of the environmental isolate improves relevance, but dual-species models simplify natural processing communities.
Rodrigues dos Santos et al., 2023	Constructed biofilm of *L. monocytogenes*, *Salmonella typhimurium*, and *Pseudomonas aeruginosa* on polypropylene.	Defined three-species community; laboratory intervention study.	Untreated multispecies biofilms were compared with black pepper essential oil and piperine treatments; sessile-cell counts and biofilm-related gene expression were evaluated over time.	Shows that phytochemical treatment can reduce multispecies biofilms and alter biofilm-associated gene expression. Useful mechanistic/control evidence, but the defined consortium and controlled exposure limit direct industrial extrapolation.
Kaushik et al., 2026	Dairy-related model; mono- and dual-species *Escherichia coli* and *Salmonella Typhimurium* biofilms in milk.	Defined dual-species community; laboratory treatment and predictive-modeling study.	Quercetin alone and ultrasound-assisted quercetin were evaluated across treatment conditions; RSM and GA–ANN were used to model/optimize biofilm reduction.	Dual-species biofilms were more resistant than monospecies systems, while optimized ultrasound–quercetin treatment produced strong reductions. Promising food-matrix evidence, but validation on natural industrial communities and processing surfaces remains needed.
Valiei et al., 2024	Conceptual gut-mucosal bacterial community model examining spatial organization and metabolic interactions.	Non-food, *in silico* theoretical model; complementary mechanistic evidence.	Agent-based modeling was used to explore how metabolic interactions such as competition, commensalism, and mutualism shape community structure and coexistence; no food-industry intervention.	Provides conceptual support for interaction-driven emergent spatial structure. Its value for this review is mechanistic rather than directly translational because the model is non-food and theoretical.

## 4 Implications for food safety and quality

Mixed-species biofilms can affect food production by modifying the persistence, dissemination, and behavior of pathogenic and spoilage microorganisms, with potential consequences including recurrent contamination, reduced sanitation efficacy, food spoilage, and shortened shelf life. However, the magnitude and direction of these effects are strongly context dependent and cannot always be predicted from single-species observations. The following sections examine their principal implications for persistence and cross-contamination, food quality, and public health, while considering the strength and translational limitations of the available evidence.

### 4.1 Persistence and cross-contamination in food-processing environments

A major consequence of mixed-species biofilms in food-processing environments is their potential to act as persistent or recurrent sources of contamination. Diverse microbial populations, including environmental microorganisms, spoilage bacteria, starter-associated microbiota, and foodborne pathogens, may colonize food-contact and non-food-contact surfaces, particularly protected or difficult-to-clean niches (Giaouris et al., 2015).

Mixed-species communities may exhibit greater tolerance to environmental stresses and sanitation than corresponding single-species biofilms, although this effect is not universal and depends on community composition and environmental conditions (Flemming et al., 2016). Consequently, persistence within a mixed-species biofilm cannot be inferred solely from monoculture behavior, particularly when translating findings from defined laboratory consortia to naturally occurring industrial communities.

*Listeria monocytogenes* provides a particularly relevant example. Recurrent detection over extended periods has been reported in food-processing environments; however, recurrent detection alone does not demonstrate persistence of the same strain unless supported by strain-resolved longitudinal evidence. Where strain-level persistence has been demonstrated, prolonged survival may reflect intrinsic stress tolerance together with interactions with resident microbiota (Fagerlund et al., 2017).

Observational studies have identified multispecies microbial communities associated with recurrent contamination across food sectors. Dairy-processing environments may contain *L. monocytogenes*, *Pseudomonas*, *Staphylococcus*, and lactic acid bacteria (Diaz et al., 2025), while meat-processing facilities frequently harbor *Pseudomonas* spp., *Brochothrix thermosphacta*, and, in some cases, foodborne pathogens (Wagner et al., 2020; Nikolaev et al., 2022; Yang et al., 2024; Calhoun et al., 2025). Comparable contamination reservoirs have been reported in seafood-processing, ready-to-eat, and fresh-produce facilities (Zhang et al., 2024; Carpentier and Cerf, 2011). However, community detection or profiling does not necessarily demonstrate coexistence within structurally organized mixed-species biofilms and should therefore be regarded as complementary to, rather than equivalent to, direct biofilm characterization.

Biofilm-associated contamination becomes particularly relevant when cells released through dispersal or mechanical disturbance are transferred to food products or downstream equipment. Processing and packaging equipment can thereby contribute to microbial dissemination along production chains (Fagerlund et al., 2017; EFSA Biohaz Panel, 2024). Intermittent biofilm-associated release may also complicate environmental monitoring and source attribution because contamination can remain undetected between sampling events (Tuytschaever et al., 2023).

Taken together, available evidence supports an association between resident surface communities, biofilm formation, and recurrent contamination, although the strength of causal evidence varies among studies. Longitudinal strain-resolved surveillance combined with direct characterization of biofilm architecture and microbial activity is therefore needed to distinguish true persistence from repeated introduction and to establish the contribution of mixed-species biofilms to cross-contamination under industrial conditions.

### 4.2 Impact on food spoilage and shelf life

Mixed-species biofilms can affect food quality by providing reservoirs of spoilage microorganisms that may be repeatedly introduced into products during processing. Microbial communities developing within food matrices also contribute to spoilage through ecological succession and metabolic interactions, but these systems should not be considered equivalent: surface-associated biofilms represent potential contamination sources, whereas food-associated microbial consortia provide complementary evidence for understanding community-level processes underlying spoilage.

Culture-independent studies indicate that spoilage frequently results from dynamic microbial consortia rather than from a single dominant organism. Microbial interactions and succession can alter both the rate and nature of quality deterioration, producing outcomes that differ from monoculture-based predictions (Ercolini et al., 2015). Depending on community composition and environmental conditions, these interactions may accelerate, delay, or redirect spoilage.

Sector-specific studies illustrate this variability. In meat and meat products, communities containing *Pseudomonas* spp., *Brochothrix thermosphacta*, and Enterobacteriaceae contribute to off-odors, discoloration, slime formation, and textural deterioration (Calhoun et al., 2025). In seafood, *Pseudomonas* spp., *Shewanella* spp., and *Photobacterium phosphoreum* contribute to the production of trimethylamine, sulfur compounds, biogenic amines, and other spoilage metabolites (Gram and Dalgaard, 2002; Parlapani et al., 2014; Sheng and Zhu, 2021). In dairy systems, interactions among starter and non-starter microorganisms may contribute positively to flavor and texture development but can also contribute to off-flavors, gas production, and bitterness (Mayo et al., 2021; Blaya et al., 2018). Additional spoilage defects include late blowing associated with butyric acid-producing *Clostridium* spp. (Qian et al., 2022). Fresh and minimally processed produce likewise supports complex microbial communities associated with tissue degradation, softening, discoloration, and reduced shelf life (Brandl, 2006). Biofilm-forming bacteria have also been recovered from equipment surfaces, wash water, and products in industrial fresh-produce processing, highlighting the potential contribution of persistent microbial reservoirs to spoilage and sanitation challenges (Langlois et al., 2026).

Preservation and storage conditions further shape these ecological relationships. Refrigeration, modified-atmosphere packaging (MAP), vacuum packaging, and washing impose selective pressures that alter community succession and favor microorganisms adapted to specific conditions. Such preservation and storage conditions are important determinants of microbial spoilage and shelf life (Karanth et al., 2023), while changes in community composition under MAP have been associated with differences in spoilage development and metabolite production (Cha et al., 2023). Persistent surface-associated communities provide an additional link between processing-environment ecology and product quality by repeatedly introducing spoilage microorganisms into foods, potentially contributing to reduced shelf life despite preservation and sanitation measures (EFSA Biohaz Panel, 2024). However, direct quantitative evidence linking specific industrial mixed-species biofilms to subsequent changes in product shelf life remains limited. Consequently, quantitative spoilage rates or shelf-life reductions reported for food-associated microbial communities cannot necessarily be attributed to mixed-species biofilm reservoirs without direct source-to-product evidence.

Overall, spoilage is best understood as a community-level process shaped by microbial interactions, environmental selection, and succession. Distinguishing the contribution of surface-associated biofilm reservoirs from microbial dynamics occurring within food matrices remains essential for translating ecological knowledge into interventions aimed at reducing spoilage and extending shelf life.

### 4.3 Public health implications

Mixed-species biofilms may influence public health by modifying the survival, persistence, and dissemination of foodborne pathogens. Incorporation into established microbial communities can alter pathogen responses to environmental stress and sanitation, but these effects depend on species composition, biofilm architecture, environmental conditions, and developmental stage. Consequently, increased survival observed in laboratory consortia should not be generalized to all mixed-species biofilms.

Biofilm-associated protection can enhance pathogen survival under environmental stresses and sanitation, although antagonistic interactions may produce the opposite outcome (Bridier et al., 2011; Flemming et al., 2016). Food-associated studies indicate that resident microbiota can influence the survival of pathogens such as *L. monocytogenes* and *Salmonella enterica* (Kim et al., 2023), whereas evidence for STEC and *Campylobacter jejuni* in naturally occurring industrial mixed-species biofilms remains less extensive. This variability reinforces the need to distinguish mechanistic evidence from defined consortia from direct observations in naturally occurring industrial communities.

A further public health concern is the potential contribution of biofilm-associated communities to antimicrobial resistance (AMR). Genetically acquired resistance must be distinguished from biofilm-associated phenotypic tolerance, which is generally reversible and may result from altered growth, physiological heterogeneity, stress responses, protected microniches, or reduced antimicrobial exposure. Persister cells represent a further distinct phenomenon, comprising transiently tolerant subpopulations that survive antimicrobial exposure without necessarily carrying resistance determinants (Hall and Mah, 2017; Serrano et al., 2025). Thus, reduced antimicrobial susceptibility within a biofilm should not itself be interpreted as genetically encoded resistance.

Repeated exposure to sublethal sanitizer concentrations may promote physiological adaptation or select populations with reduced susceptibility, although such responses should be distinguished from stable, heritable resistance. Biofilm-associated communities may also provide conditions favorable to horizontal gene transfer (HGT), as demonstrated in experimental models, including through natural transformation (Ma et al., 2021). Metagenomic analyses of slaughterhouse, dairy, and meat-processing environments have identified diverse antimicrobial resistance genes and mobile genetic elements (Alvarez-Molina et al., 2023); however, their presence does not demonstrate active HGT within naturally occurring mixed-species biofilms. Direct food-specific evidence remains limited, and food-associated biofilms should therefore be regarded as environments potentially conducive to gene exchange rather than automatically characterized as AMR “hotspots” (Rossi et al., 2014).

Ready-to-eat (RTE) foods warrant particular attention because they are consumed without a subsequent processing step specifically intended to eliminate microbial contamination, allowing persistent environmental reservoirs to provide a potential route to consumer exposure. This is particularly relevant for L. monocytogenes, which has been associated with outbreaks involving deli meats, smoked fish, soft cheeses, and fresh produce. Vulnerable populations may experience severe outcomes following exposure to L. monocytogenes, STEC, Salmonella spp., Cronobacter sakazakii, and other foodborne pathogens (EFSA Biohaz Panel, 2018).

Persistent contamination can also generate substantial economic and societal costs through recalls, production interruptions, intensified monitoring, and outbreak investigation. However, these burdens reflect foodborne contamination broadly and should not be attributed specifically to mixed-species biofilms without a demonstrated epidemiological link.

Collectively, available evidence indicates that mixed-species biofilms can modify pathogen persistence, antimicrobial susceptibility, contamination dynamics, and potentially the dissemination of resistance determinants. However, evidence strength differs among these outcomes: mechanistic effects are often well demonstrated in laboratory biofilm models, whereas direct causal links among specific industrial mixed-species communities, product contamination, consumer exposure, and disease remain more difficult to establish. Integrating ecological information with longitudinal environmental surveillance, strain-resolved pathogen tracking, and quantitative exposure assessment will therefore be essential for determining when community-level processes translate into measurable public health risk. These relationships are summarized in Table 5.

**TABLE 5:** Ecological processes linking mixed-species biofilms to food safety and quality outcomes.

Ecological process	Persistence & cross-contamination	Food spoilage & shelf life	Public health
Cooperation	Matrix stabilization, stress protection	Cross-feeding, metabolite production	Enhanced pathogen persistence
Competition	Selection of persistent populations	Succession of spoilage microbiota	Colonization resistance or pathogen selection
Communication	Coordinated dispersal	Collective metabolic regulation	Virulence modulation
Spatial organization	Protected microniches	Functional heterogeneity	Reduced sanitizer penetration
Emergent properties	Long-term reservoirs	Accelerated quality deterioration	Increased contamination risk

## 5 Control and management of mixed-species biofilms

The structural and ecological complexity of mixed-species biofilms can limit the extrapolation of results obtained with planktonic cells or single-species biofilms to naturally occurring food-processing communities. Effective management therefore requires integrating conventional hygiene practices with complementary interventions targeting specific stages or processes involved in biofilm establishment, persistence, and recolonization. Such approaches should complement rather than replace established sanitation procedures, with their applicability determined by evidence strength, technological maturity, safety, and operational feasibility. The principal ecological targets and representative management strategies are summarized in Table 6.

**TABLE 6:** Ecology-informed management strategies for mixed-species biofilms in food systems.

Ecological insight	Management target	Representative intervention	Evidence level	Industrial maturity	Main limitations / safety and regulatory considerations
Environmental filtering	Prevent surface colonization	Hygienic design, surface engineering, environmental monitoring	High for hygienic design and routine monitoring; moderate for advanced surface engineering	High for hygienic design and monitoring; application-specific/emerging for engineered surfaces	Performance depends on equipment geometry, material properties, processing conditions, maintenance, and sanitation compatibility. Novel surface treatments may require assessment of durability, migration, food-contact safety, and regulatory compliance.
Community assembly	Early colonizers and influential community members	Protective cultures, competitive exclusion, targeted ecological replacement	Moderate mechanistic and food-related evidence; limited direct validation in complex industrial biofilms	Application-specific	Effects are strain-, community-, and environment-dependent. Requires evaluation of strain safety, ecological fitness, stability, technological compatibility, unintended community effects, and regulatory acceptance.
Cooperation	EPS production and metabolic cross-feeding	Matrix-degrading enzymes, dispersal agents	Moderate experimental evidence; industrial evidence remains limited	Emerging to application-specific	Efficacy depends on matrix composition and community structure. Enzyme stability, delivery, contact time, formulation, cost, compatibility with processing conditions, and potential effects on food-contact materials require validation.
Communication	Quorum sensing and interspecies signaling	Quorum-quenching enzymes, signaling inhibitors	Limited-to-moderate mechanistic evidence; comparatively limited evidence in representative food-associated mixed-species biofilms	Emerging	Strong species- and context-dependence; signaling inhibition may not uniformly reduce biofilm formation. Delivery, stability, persistence of activity, process integration, and regulatory feasibility remain major constraints.
Competition	Community structure and niche occupation	Competitive exclusion, targeted microbial modulation, ecological engineering approaches	Moderate mechanistic evidence; limited direct industrial validation	Emerging / application-specific	Outcomes depend on colonization history, resident microbiota, environmental conditions, and target organisms. Reproducibility across facilities may be limited, and intentional community manipulation requires careful safety and regulatory assessment.
Spatial organization	Matrix architecture and protected microenvironments	Optimized cleaning, enzymatic sanitation, improved disinfectant penetration	Moderate-to-high mechanistic evidence; variable validation under food-processing conditions	Moderate to high for optimized sanitation; application-specific for enzymatic approaches	Effectiveness depends on biofilm maturity, matrix composition, surface geometry, physicochemical gradients, and disinfectant compatibility. Validation under realistic flow, residue, temperature, and surface conditions is required.
Emergent properties	Community resilience, persistence, and sanitation tolerance	Multi-target strategies combining chemical, enzymatic, biological, or physical interventions	Moderate experimental evidence; industrial evidence remains heterogeneous	Emerging / application-specific	Combined strategies may improve efficacy but increase operational complexity. Scalability, compatibility, cumulative safety, cost, reproducibility, and regulatory requirements must be evaluated case by case.
Ecological succession	Recolonization dynamics and recurrent contamination	Continuous monitoring, predictive microbiology, adaptive sanitation programs	Moderate for longitudinal monitoring; limited-to-moderate for ecological prediction	Moderate for monitoring; emerging for predictive/adaptive ecological control	Predictive reliability depends on standardized, longitudinal, representative datasets and facility-specific validation. Data interoperability, calibration, actionable thresholds, and integration into existing hygiene systems remain important challenges.

Information summarized from Flemming et al. (2016); Bridier et al. (2015); Nadell et al. (2016); Røder et al. (2016); Mukherjee and Bassler (2019); Srey et al. (2013); Simões et al. (2010); Sadiq et al., 2023a,b; Butaitë et al. (2017); Valiei et al. (2024); Jeong and Kim (2025); Calhoun et al. (2025); Bäcker et al. (2026); Coppola et al. (2025b); Tremonte et al. (2017).

### 5.1 Limitations of conventional sanitation approaches

Cleaning and disinfection remain fundamental components of microbial control in food-processing environments. Cleaning-in-place (CIP), mechanical cleaning, and chemical disinfectants effectively reduce surface contamination, but established biofilms may remain incompletely removed or inactivated by conventional cleaning and sanitation procedures (Pant et al., 2023). Matrix properties, biofilm architecture, physiological heterogeneity, and interspecies interactions can modify treatment susceptibility and permit survival of particular populations (Simões et al., 2010; Bridier et al., 2015). Repeated exposure to sublethal disinfectant concentrations resulting from inadequate cleaning, insufficient contact time, organic fouling, or inaccessible niches may promote stress adaptation or favor populations with reduced susceptibility (Flemming et al., 2016; Møretrø et al., 2017; Nordholt et al., 2021). Such responses should not automatically be interpreted as stable disinfectant resistance but may facilitate survival and recolonization following sanitation.

Effective sanitation therefore depends not only on disinfectant selection but also on organic-soil removal, equipment accessibility, hydrodynamics, and hygienic design. Protected or difficult-to-clean niches can retain organic material and residual microorganisms and contribute to recolonization (Bremer et al., 2006). Sequential approaches combining cleaning, matrix disruption, and microbial inactivation may improve efficacy, provided that detached cells are subsequently removed or inactivated (Lequette et al., 2010; Palafox-Rivera et al., 2025). Because treatment susceptibility varies among communities and processing environments (Giaouris et al., 2014), these limitations support precision sanitation approaches adapted to contamination history, microbial ecology, and facility-specific operational characteristics.

### 5.2 Complementary strategies targeting mixed-species biofilms

A range of complementary approaches has been investigated to improve the prevention, disruption, or removal of mixed-species biofilms, including enzymatic treatments, bacteriophages, plant-derived antimicrobials, protective microorganisms, biosurfactants, nanotechnology, surface engineering, and quorum quenching. Their evidence base and technological maturity vary substantially. While some approaches have been evaluated using food-associated mixed-species biofilms, much of the available evidence still derives from defined laboratory systems, and comparatively few technologies have undergone extensive pilot-scale or industrial validation. These differences should be considered when assessing their translational potential.

#### 5.2.1 Enzymatic treatments

Proteases, glycosidases, DNases, and dispersin-like enzymes target components of the extracellular matrix, weakening biofilm cohesion and potentially improving antimicrobial penetration. Because mixed-species matrices contain polymers produced by different microorganisms, combinations of enzymes may provide broader matrix disruption than individual treatments (Simões et al., 2010; Flemming et al., 2016). Matrix degradation alone, however, does not ensure microbial inactivation, and detached viable cells may retain the capacity to recolonize surfaces. Enzymatic treatments are therefore most appropriately considered as components of sequential or combined sanitation procedures (Lequette et al., 2010; Palafox-Rivera et al., 2025).

#### 5.2.2 Bacteriophages

Bacteriophages provide highly selective activity against susceptible bacterial hosts, while phage-derived depolymerases may facilitate disruption of extracellular polysaccharides. Antibiofilm activity has been reported against major foodborne pathogens including *L. monocytogenes*, *Salmonella enterica*, and Shiga toxin-producing *Escherichia coli* (Alian et al., 2026; Gutiérrez et al., 2016). However, narrow host range represents an important limitation in taxonomically diverse mixed-species biofilms, and community composition may leave non-susceptible populations unaffected. Phage cocktails and combinations with enzymes, disinfectants, or other antimicrobials may broaden activity, but routine industrial implementation requires validation of host coverage, resistance development, production consistency, formulation and stability, regulatory compliance, and efficacy under realistic processing conditions. Thus, phage-based approaches appear more readily applicable to targeted control of defined bacterial hazards than to broad-spectrum elimination of complex mixed-species biofilms.

#### 5.2.3 Plant-derived antimicrobials

Plant extracts, essential oils, polyphenols, terpenes, and natural bioactive proteins such as lactoferrin can interfere with microbial adhesion, cellular integrity, matrix production, and biofilm development through multiple mechanisms (Ayala-Zavala et al., 2014; Quintero-Martínez et al., 2024). Their antimicrobial and antibiofilm activities against food-associated microorganisms are supported by substantial laboratory evidence (Uddin Mahamud et al., 2024; Khammassi et al., 2022; Nazzaro et al., 2022; Luna-Solorza et al., 2023; Monteiro Marques et al., 2023; Rodrigues dos Santos et al., 2023; Nilofar et al., 2024; De Rossi et al., 2025; Khedhri et al., 2025), including studies involving mixed-species systems (Ayed et al., 2024). Translation to industrial application remains more challenging because activity depends on composition, concentration, target community, surface or food matrix, and environmental conditions. Batch-to-batch standardization, chemical stability, sensory effects where food contact is involved, material compatibility, scalable formulation and delivery, and regulatory acceptance therefore require case-specific evaluation. Accordingly, strong laboratory antibiofilm activity should not be interpreted as evidence of equivalent performance under industrial processing conditions.

Honey and honey-derived phytocomplexes represent additional multitarget natural antimicrobials with reported antibiofilm activity (Nazzaro et al., 2024; Coppola et al., 2025a). Physical technologies such as ultrasound may enhance the activity of natural compounds or conventional disinfectants by facilitating biofilm disruption and antimicrobial penetration (Shao et al., 2020; Kaushik et al., 2026). However, the efficacy, scalability, technological compatibility, and potential sensory consequences of such combined approaches require validation under application-specific industrial conditions.

#### 5.2.4 Protective microorganisms

Beneficial microorganisms can interfere with pathogen or spoilage-biofilm establishment through competition for nutrients and attachment sites, modification of local environmental conditions, and production of antimicrobial metabolites. Lactic acid bacteria, for example, can produce organic acids and other antimicrobial metabolites, including 3-phenyllactic acid, with inhibitory activity against foodborne microorganisms (Sorrentino et al., 2018). Their use as protective cultures or ecological competitors is promising, but application requires careful assessment of strain safety, technological compatibility, ecological fitness, and behavior within resident microbial communities.

#### 5.2.5 Biosurfactants

Microbial biosurfactants can reduce adhesion by modifying surface physicochemical properties and may also destabilize established biofilms (Datta and Bhattacharjee, 2025). However, their effects vary with surface characteristics, microbial composition, concentration, and processing conditions, and evidence from naturally occurring food-industry mixed-species biofilms remains comparatively limited.

#### 5.2.6 Nanotechnology

Metallic and metal-oxide nanoparticles and nanostructured delivery systems can exert antimicrobial and antibiofilm effects through multiple mechanisms and may improve the stability or delivery of active compounds within biofilms (Landa et al., 2025; Nguyen, 2026). Despite promising proof-of-concept results, translation to food-processing applications remains constrained by the need to establish toxicological safety, potential migration or environmental release, regulatory acceptability, material compatibility, scalability, and long-term performance under repeated operational use. Consequently, nanotechnology should currently be regarded primarily as an emerging platform requiring further application-specific validation rather than as a broadly deployable alternative to established sanitation.

#### 5.2.7 Surface engineering

Anti-adhesive, microtextured, photocatalytic, and antimicrobial surfaces seek to prevent or delay microbial attachment before mature biofilms become established (Elbehiry and Alajaji, 2026; Hindieh et al., 2025). Their preventive character distinguishes them from strategies directed at established biomass. However, fouling, surface wear, repeated sanitation, and changes in material properties may reduce performance over time, making long-term validation under operational conditions essential.

#### 5.2.8 Quorum quenching

Quorum-quenching approaches interfere with microbial signaling through inhibition of signal production or perception or through enzymatic signal degradation (Grandclément et al., 2015; Zhao et al., 2026; Papaneophytou, 2026). Because signaling networks can influence matrix production, motility, stress responses, and dispersal, their disruption may modify biofilm development without necessarily exerting direct bactericidal activity. However, signaling systems differ among species, and responses to quorum quenching can vary substantially with community composition and environmental conditions. Evidence from representative food-associated mixed-species biofilms remains comparatively limited, while practical implementation also requires consideration of delivery, stability, persistence of activity, process compatibility, and regulatory feasibility (Jadhav, 2026). Quorum quenching should therefore presently be viewed as a promising complementary strategy requiring further validation rather than as a stand-alone replacement for sanitation.

Taken together, these technologies should be regarded as complementary tools rather than universally effective alternatives to established hygiene practices. Their translational potential varies considerably, from approaches that can already complement targeted sanitation under defined conditions to technologies that remain predominantly at laboratory or proof-of-concept stage. Practical adoption will depend on matching the intervention to the target community, surface, processing conditions, and stage of biofilm development, while considering evidence strength, scalability, safety, regulatory feasibility, operational compatibility, and cost. These dimensions are summarized in Table 6.

### 5.3 Toward ecologically informed biofilm management

Ecology-informed management builds on conventional sanitation by using knowledge of community assembly, contamination history, microbial interactions, and recolonization dynamics to determine where, when, and how interventions should be applied. Its purpose is not to preserve biofilms or replace microbial inactivation, but to identify ecological vulnerabilities that can improve the effectiveness and durability of established hygiene measures. Potential targets include pioneer colonizers, matrix-producing populations, metabolic dependencies, and critical stages of community development. *Pseudomonas* spp., for example, may act as early colonizers or contribute disproportionately to matrix formation and community development in food-processing environments (Wagner et al., 2020; Calhoun et al., 2025), while particular interspecies dependencies may influence community stability (Sadiq et al., 2023b; Bäcker et al., 2026). However, such roles are context dependent, and taxa identified as influential in one facility or experimental consortium should not automatically be regarded as universal keystone organisms.

Timing and contamination history are equally important. Monitoring recolonization after sanitation may help identify recurrently colonized surfaces and inform the timing of targeted interventions (Fagerlund et al., 2017). At facility level, ecology-informed management requires integration of environmental monitoring, contamination history, processing conditions, equipment design, and, where informative, microbial community data. Distinguishing persistent reservoirs from repeated external introduction is particularly important because the two scenarios imply different interventions: persistent niches may require equipment redesign or targeted intensive sanitation, whereas repeated introductions may be better addressed through segregation, raw-material control, or source management (Fagerlund et al., 2017). Community profiling and predictive models may complement routine culture-based surveillance by identifying recurrent ecological patterns or high-risk locations, but their practical value depends on standardized datasets and validation against measurable contamination outcomes (Carpentier and Cerf, 2011).

Current evidence therefore supports an integrated rather than replacement-based model of biofilm management. Conventional cleaning and disinfection remain indispensable, while ecological information may improve the targeting of interventions, environmental surveillance, and post-treatment verification. However, ecology-informed management should currently be regarded primarily as a decision-support framework rather than as a standardized industrial control technology. Its broader implementation will depend on identifying ecological indicators that provide reproducible and actionable information across real processing environments. As discussed in Section “6.4 Limitations of the current evidence”, this will require standardized multispecies models, longitudinal and strain-resolved industrial studies, pilot-scale validation, and explicit consideration of safety, regulatory feasibility, operational compatibility, and cost.

## 6 Future perspectives and research needs

Significant advances have improved our understanding of the structure, interactions, and persistence of mixed-species biofilms in food systems. However, translating this knowledge into predictive and operational tools remains challenging. Future research should increasingly connect mechanistic understanding with food-relevant experimental models, longitudinal environmental surveillance, quantitative risk assessment, and validated intervention strategies. Emerging analytical and computational approaches may support this transition by identifying measurable ecological signatures of biofilm establishment, persistence, and recolonization, although their technological maturity and proximity to routine industrial application vary considerably (Coppola et al., 2025b; Dutta et al., 2024). Figure 3 summarizes this integrated perspective, in which conventional control, ecology-informed management, and predictive approaches operate as complementary components rather than sequential alternatives.

**FIGURE 3:**
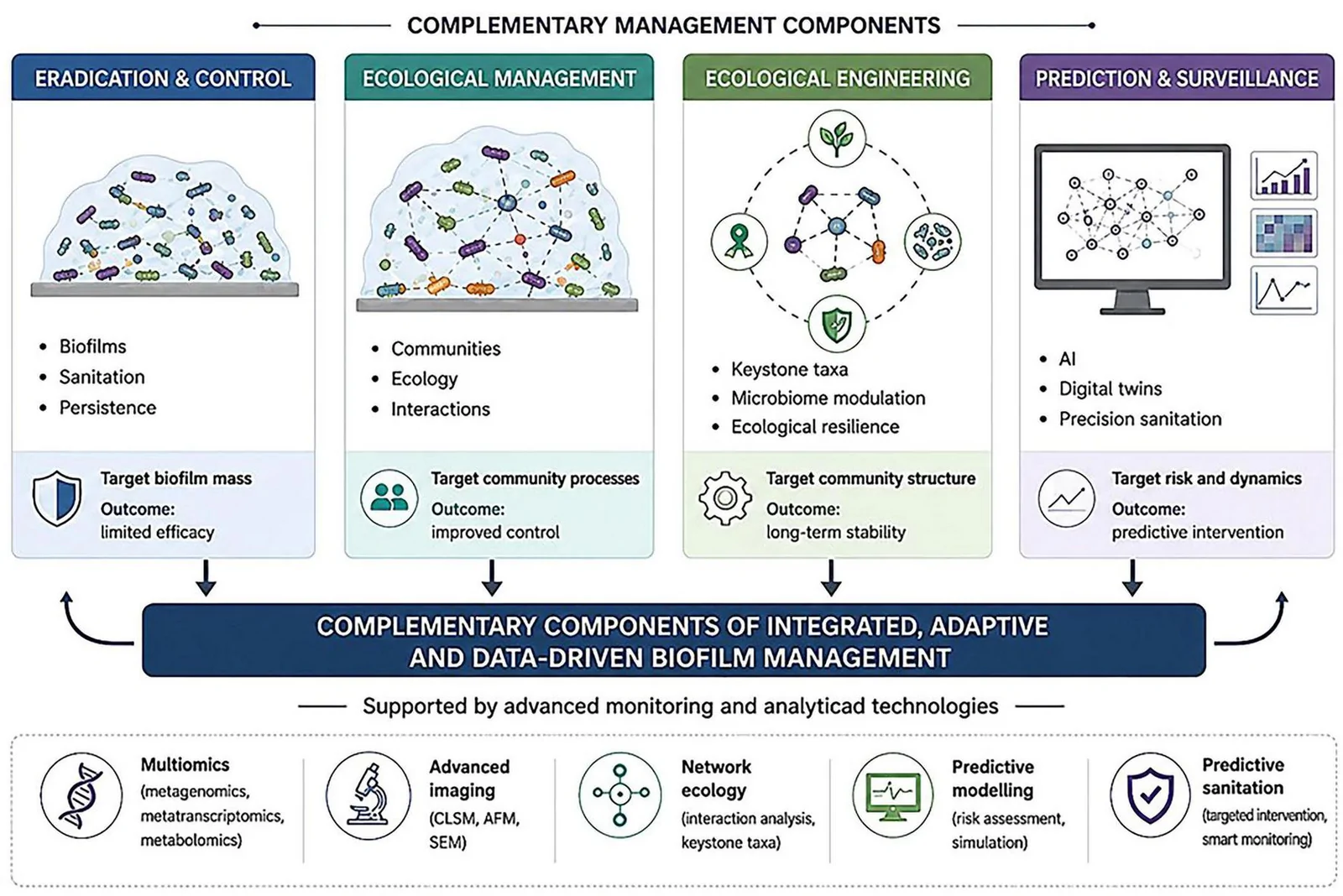
Integrated framework for mixed-species biofilm management combining conventional control, ecology-informed management, and predictive approaches.

### 6.1 Emerging technologies and food-relevant experimental models for mixed-species biofilm investigation

A major challenge in studying food-associated mixed-species biofilms is resolving microbial interactions within structurally complex and dynamic communities. Conventional culture-based methods remain essential for quantifying cultivable populations but cannot alone resolve spatial organization, physiological heterogeneity, or functional interactions. Advanced imaging approaches can complement these methods by providing spatially resolved information: FISH combined with confocal laser scanning microscopy (CLSM), for example, enables taxonomic identification within three-dimensional biofilm architecture, while SEM, AFM, imaging mass spectrometry, and Raman-based techniques provide complementary structural or biochemical information (Rodrigues et al., 2023).

Integration with metagenomics, metatranscriptomics, metaproteomics, and metabolomics can link community composition with gene expression, metabolic activity, and spatial organization (Quince et al., 2017; Knight et al., 2018). The value of these approaches lies not simply in generating increasingly complex datasets, but in identifying reproducible functional relationships and measurable community features associated with persistence, stress responses, or contamination outcomes.

Experimental realism remains equally important. Much mechanistic knowledge derives from single-species biofilms or defined laboratory consortia, which are valuable for establishing causal relationships but incompletely reproduce the complexity of food-processing facilities (Røder et al., 2016; Kim et al., 2023). Food-relevant models should therefore complement reductionist systems by incorporating representative microbial communities, processing surfaces, food residues, and relevant environmental and sanitation conditions (Fagerlund et al., 2017). Microfluidic devices and biofilm-on-chip platforms may help bridge these levels of complexity by allowing controlled manipulation of environmental variables while retaining spatial and temporal resolution.

The objective should therefore not be to replace simplified experimental systems, but to establish complementary levels of evidence ranging from mechanistically tractable consortia to food-relevant models, pilot-scale systems, and naturally occurring industrial communities (Røder et al., 2016). Such integration is necessary to determine which interactions identified under controlled conditions remain relevant during food processing. In the near term, priority should be given to standardized food-relevant models and measurable processes such as early colonization, recolonization, and sanitation response, which can provide experimentally tractable links between community ecology and persistence.

### 6.2 Predicting biofilm development and adaptive management

A major research priority is to determine whether microbial and environmental information can predict the transition from transient surface contamination to persistent biofilm establishment. Candidate predictors include early community composition, colonization history, environmental conditions, spatial organization, and populations that disproportionately influence community development (Garza et al., 2026). However, their predictive value is likely to be context dependent and should be validated across communities, facilities, and processing conditions rather than inferred from individual experimental systems.

In the near term, the most realistic opportunity lies in integrating longitudinal environmental monitoring with standardized microbial and operational data to identify reproducible indicators of recurrent contamination or biofilm establishment. High-throughput microbiome and multi-omics analyses may complement conventional surveillance when microbial signatures can be linked to measurable contamination outcomes (Xu et al., 2025; Debode et al., 2024). Machine-learning approaches may assist in integrating these heterogeneous datasets, but their predictive reliability depends on sufficiently large, standardized, representative, and prospectively validated datasets (Taiwo et al., 2024). Thus, their immediate value is likely to lie primarily in decision support and hypothesis generation rather than autonomous prediction of industrial biofilm behavior.

Over a longer time horizon, ecological forecasting may provide frameworks for identifying early-warning signals associated with shifts toward persistent contamination (Dietze et al., 2018). Digital-twin approaches are already being explored in food processing for real-time process simulation and control (Kannapinn et al., 2022), and their future extension to microbiological, environmental, and operational data could provide a framework for simulating contamination dynamics and comparing intervention scenarios. These applications remain emerging concepts for food-associated biofilm management and require prospective validation against real contamination events before they can support routine operational decisions. Their development will depend not only on model performance but also on data standardization, interoperability, facility-specific calibration, and the ability to generate reproducible and actionable predictions.

Validated predictive information could ultimately support adaptive management, in which intervention intensity, timing, and location are informed by contamination history, facility characteristics, environmental surveillance, and microbial indicators. Such approaches should complement rather than replace standardized hygiene programs, directing additional interventions toward locations or periods associated with elevated contamination risk.

### 6.3 Incorporating mixed-species biofilm dynamics into food safety risk assessment

Future research should bridge current understanding of mixed-species biofilms with quantitative microbial risk assessment (QMRA). Existing frameworks primarily assess hazards at the level of individual microorganisms, whereas contamination in food-processing environments may involve complex surface-associated communities. Incorporating mixed-species biofilm dynamics could improve representation of contamination processes, but only where ecological observations can be translated into measurable model parameters. Relevant variables may include biofilm growth and dispersal, surface-to-food transfer, pathogen abundance within mixed communities, sanitation-related log reductions, and recolonization following treatment.

Some biofilm-related variables are already compatible with quantitative risk-assessment approaches. Surface-to-food transfer of biofilm-associated cells, for example, has been considered within QMRA frameworks for *Listeria monocytogenes* processing chains, illustrating how environmental contamination could be linked quantitatively to product exposure (Tomasello et al., 2024). Longitudinal environmental monitoring could similarly provide estimates of recurrent contamination or recolonization, while community profiling may identify candidate microbial or environmental indicators associated with pathogen abundance or biofilm establishment.

For incorporation into QMRA, however, microbial or ecological indicators must be quantitatively linked to product contamination and ultimately to consumer exposure through parameters such as transfer probabilities, contamination frequencies, pathogen concentrations, and subsequent growth or survival. These relationships are likely to vary among facilities, food sectors, surfaces, sanitation regimes, and microbial communities, requiring explicit treatment of biological variability and parameter uncertainty. Resident-community and microbiome data may improve identification of persistent contamination reservoirs (Tuytschaever et al., 2023; Xu et al., 2025), but their value for risk assessment depends on demonstrating reproducible relationships with pathogen occurrence or transfer. This is particularly relevant for ready-to-eat foods, where environmental persistence and contamination routes are recognized components of *L. monocytogenes* risk (EFSA Biohaz Panel, 2018; Al-Bulushi et al., 2026).

Potential indicators such as recurrent contamination patterns, shifts in community composition, or microbial signatures associated with sanitation failure could contribute to risk models if reproducibly linked to pathogen transfer or product contamination (Debode et al., 2024; Xu et al., 2023). Ecological descriptors should therefore not be incorporated into QMRA solely as qualitative information; their predictive value requires quantitative relationships with measurable contamination outcomes (Hamilton et al., 2024; Hamilton et al., 2025; Klümper et al., 2025). In the near term, priority should be given to biofilm-related parameters that can already be measured and validated within existing risk-assessment frameworks. More complex community-level descriptors may ultimately support dynamic risk models, but their routine incorporation will require standardized measurements and validation across facilities, food sectors, and processing conditions.

### 6.4 Limitations of the current evidence

Despite substantial advances in the study of mixed-species biofilms, several limitations constrain the interpretation and translation of current evidence. Mechanistic knowledge still relies heavily on defined laboratory consortia, which provide experimental control but incompletely reproduce the taxonomic diversity, spatial heterogeneity, environmental fluctuations, and disturbance regimes of food-processing facilities. Conversely, industrial studies provide greater ecological realism but are frequently observational and therefore offer more limited causal resolution. These complementary strengths and limitations should be considered when extrapolating findings across experimental and industrial systems.

Methodological heterogeneity represents a further limitation. Differences in community composition, inoculum size, surface material, growth conditions, biofilm age, sanitation protocols, and analytical endpoints complicate comparisons among studies and may contribute to apparently contradictory findings. Because microbial interactions can change with environmental conditions, spatial organization, and colonization history, effects observed in one experimental system should not be assumed to occur similarly across food sectors or processing environments.

Industrial evidence is additionally limited by frequent reliance on cross-sectional community profiling or recurrent taxon detection. Such observations can reveal associations between microbial assemblages and contamination patterns but do not, by themselves, demonstrate spatially organized mixed-species biofilms, causal ecological interactions, or persistence of the same strain over time. Longitudinal, strain-resolved studies integrating species-resolved imaging, molecular analyses, and detailed environmental metadata are therefore needed to connect community structure with persistence, sanitation response, and product contamination.

Finally, the technological maturity of emerging control, ecology-informed, and predictive approaches remains uneven. Evidence ranges from laboratory proof-of-concept studies to application-specific evaluations under more realistic conditions, but broad industrial validation remains limited for many emerging approaches. Translation therefore requires food-relevant validation, quantitative assessment of outcomes, and explicit consideration of safety, regulatory feasibility, scalability, operational compatibility, and cost. Future research should prioritize approaches that connect mechanistic insight with reproducible industrial outcomes rather than technological complexity alone.

## 7 Conclusion

Mixed-species biofilms represent an important form of microbial organization in food-processing environments, in which community assembly, microbial interactions, spatial organization, and environmental filtering can influence persistence, contamination dynamics, spoilage, and responses to sanitation. These community-level properties are strongly context dependent and cannot always be predicted from the behavior of individual microorganisms studied in isolation.

This review highlights the need to connect mechanistic knowledge with evidence obtained under increasingly food-relevant and industrial conditions. Defined laboratory consortia remain essential for establishing causal relationships, whereas reconstructed communities, pilot-scale systems, longitudinal surveillance, and direct industrial studies provide complementary levels of translational evidence.

From an applied and research perspective, four priorities emerge. First, studies should distinguish structurally organized mixed-species biofilms from broader community detection and, where persistence is inferred, support this interpretation with longitudinal and strain-resolved evidence. Second, standardized food-relevant multispecies models should be progressively linked to pilot-scale and industrial validation, with emphasis on measurable outcomes such as persistence, recolonization, sanitation response, and product contamination. Third, ecological information should complement rather than replace established cleaning, disinfection, hygienic design, and environmental monitoring, particularly by helping distinguish persistent reservoirs from repeated external introduction and by identifying critical intervention points. Fourth, microbiome-derived indicators, multi-omics data, and predictive approaches should be incorporated into industrial decision-making or quantitative microbial risk assessment only when they can be translated into reproducible, measurable, and actionable parameters and validated across facilities and food sectors.

Future progress will therefore depend less on increasing technological complexity than on generating evidence that is reproducible, quantitatively interpretable, and operationally relevant. Integrating such evidence with conventional sanitation, environmental surveillance, and quantitative risk assessment may support more targeted, adaptive, and preventive management of mixed-species biofilms while maintaining food safety as the primary objective.
